# Pilot evaluation of 3D-designed custom-made integrated mask and headgear for children using non-invasive ventilation

**DOI:** 10.1136/bmjresp-2025-003770

**Published:** 2026-07-29

**Authors:** Mollie Broadbent, Sarah Shortland, Matt Willox, Peter Metherall, Jacob Branchflower, Vanessa E Craven, Heather E Elphick

**Affiliations:** 1 University of Sheffield, Sheffield, UK; 2 Sheffield Children’s NHS Foundation Trust, Sheffield, UK; 3 Design Futures, Sheffield Hallam University, Sheffield, UK; 4 Sheffield Teaching Hospitals NHS Foundation Trust, Sheffield, UK; 5 Health-Tech Research Centre for Children and Young People, NIHR, London, UK

**Keywords:** Non invasive ventilation

## Abstract

**Objectives:**

Use of long-term non-invasive ventilation (NIV) for children is increasing. Standard masks often fit poorly, causing significant complications. This pilot study aimed to evaluate a customised NIV mask/headgear.

**Setting:**

The evaluation took place in a single tertiary care centre.

**Participants:**

Five children aged 2–15 years using NIV for respiratory support and for whom a well-fitting commercial mask could not be found completed the study. Those requiring ventilation for rescue breaths or supplementary oxygen were excluded.

**Intervention:**

Each participant received a 3D-designed NIV mask and headgear, customised to the individual using 3D scanning and computer-aided design. The custom mask and headgear were compared with the child’s usual mask and headgear for two nights each.

**Primary and secondary outcome measures:**

The primary outcome measure was comfort, measured using a 7-point Likert scale; secondary outcome measures were facial skin marks, measured using a diary card; ventilator efficacy, measured by 4% oxygen desaturation index (ODI_4_) and sleep quality, measured by actigraphy recordings of overnight ‘Actual Wake’.

**Results:**

Compared with the usual mask/headgear, the median discomfort score using the custom mask/headgear reduced from 5 Likert points (IQR 0) to 3 (IQR 2), and facial skin sores reduced from 1.5 (IQR 3) to 1 (IQR 0). Median Oxygen Desaturation Index (ODI_4_) was similar: 0.76 (IQR 0.8) with standard mask/headgear and 0.77 (IQR 0.71) with the custom mask/headgear and median Actual Wake % was 2.95 (IQR 2.31) with standard mask/headgear and 3.70 (IQR 4.85) with the custom mask/headgear.

**Conclusions:**

Customisation of NIV masks and headgear using 3D printing may provide a valuable solution for children for whom a well-fitting commercial mask cannot be found. A multicentre study will seek to clarify definitive outcomes.

**WHAT IS ALREADY KNOWN ON THIS TOPIC**

- Commercial NIV masks often fit poorly in children, causing significant adverse effects including discomfort, poor adherence, sub-optimal ventilation and facial deformity.

**WHAT THIS STUDY ADDS**

- This study demonstrates the feasibility of this promising 3D designed and printed customised NIV mask and headgear and provides valuable information with which to design a future clinical trial.

**HOW THIS STUDY MIGHT AFFECT RESEARCH, PRACTICE OR POLICY**

- 3D technology may provide a customisation solution for NIV adherence and efficacy in children. Further work including a larger-scale evaluation is needed.

## Background

 Non-invasive ventilation (NIV) is the delivery of breathing support via a facemask and headgear used to treat people whose natural breathing is ineffective. The number of children needing NIV is steadily increasing in the UK due to improved survival rates of children with complex conditions.^1^ The need for ventilation is often lifelong, and inability to ventilate eventually leads to respiratory failure and premature death. NIV has been shown to significantly improve quality of life and life expectancy of children with neuromuscular disorders,^2^ as well as reduce both hospital admissions and days spent in hospital.^3^ A good fit between the mask/headgear and the patient’s face is essential to effectively deliver the treatment, but off-the-shelf masks designed for adults do not always provide a good fit for children leading to discomfort, facial pressure sores and disturbed sleep as well as ineffective ventilation.

In an attempt to reduce air leaks caused by an ill-fitting mask, the headgear straps are often over-tightened, leading to skin breakdown and pressure sores^4^; or pressure on the growing face leading to under-development of the maxilla, mid-face flattening and malocclusion of the teeth.^5^ Early user trials by this research group highlighted that the success of a customised mask was impaired in 45% participants by the inability to find a commercially-produced headgear that held the mask on adequately.^6^ It is therefore important to consider customised headgear in addition to the mask itself.

With the advent of novel 3D technologies, several groups have attempted to produce customised masks for NIV. A recent scoping review^7^ identified 23 papers relating to the development of customised masks for NIV, many published in the last 3 years, indicating a rapidly expanding field of research. Of these, performance testing in the laboratory and healthy volunteers has yielded promising results, but only three studies involving customised cushions for adults with sleep apnoea have evaluated clinical efficacy and none have formally evaluated custom-made masks compared with standard commercial masks. The review concluded that further technical development is needed before personalised masks can be offered for NIV patients.

This pilot study hypothesised that a 3D-designed custom mask with integrated headgear would offer improvement in facial comfort as a primary measure with fewer facial skin marks, improved ventilator efficacy and improved sleep quality as secondary measures when compared with standard off-the-shelf interfaces.

## Methods

The study was a non-randomised pilot study involving children requiring breathing support with NIV for sleep-disordered breathing, for whom a well-fitting ‘off-the-shelf’ ’mask could not be found and therefore ventilation was being implemented with a poorly fitting NIV mask. Those requiring ventilation for rescue breaths or supplementary oxygen were excluded. The study received ethical approval from Liverpool Central NHS Research Ethics Committee (23/NW/037). Parents and young people were involved in the mask design, study design and dissemination of study outcomes.

A custom NIV mask and headgear were manufactured for each participant using data from 3D facial scans using a portable structured light scanner (Artec Leo | Professional 3D scanning solutions | Artec 3D). A prescription was approved by the lead clinician for each patient to comply with medical device regulations for a bespoke mask and headgear design. Nasal or oronasal masks were produced, reflecting the same mask type as the patient’s standard mask.

Pre-production 3D image data processing and production of a computer-aided design (CAD) file using Solidworks 3D CAD design software took approximately 4 hours per mask. Mask design included a 3D-printed frame covered with a silicon sheath produced by injection moulding.^8^ The headgear was made of modular parts joined together at different locations and angles, personalised to the individual. Key components included a crown strap, sized small/medium/large and a joining bar and straps which could be cut to length. 3D printed connectors affixed the fabric parts to 3D printed splitters, which provided routing around eyes and ears as well as structure to the headgear, with the aim of taking some of the load off the mask cushion. The splitters were configurable in length and angle to suit the patient. Solid parts were 3D printed using stereolithography and fused deposition modelling while the straps were produced by laser cutting and heat welding.^7^ The printing process for each mask and headgear took 2–3 days.

Participants underwent nocturnal monitoring for two nights using their standard mask and headgear (control conditions), followed by two nights using the custom mask and headgear. Participants were observed overnight by qualified staff and/or carers and safety instructions given for removal of the mask if necessary. A pre-determined threshold of 4 hours study duration was used; if the duration was less than 4 hours’ duration on any night, the data for that night were discarded.

**Figure 1:**
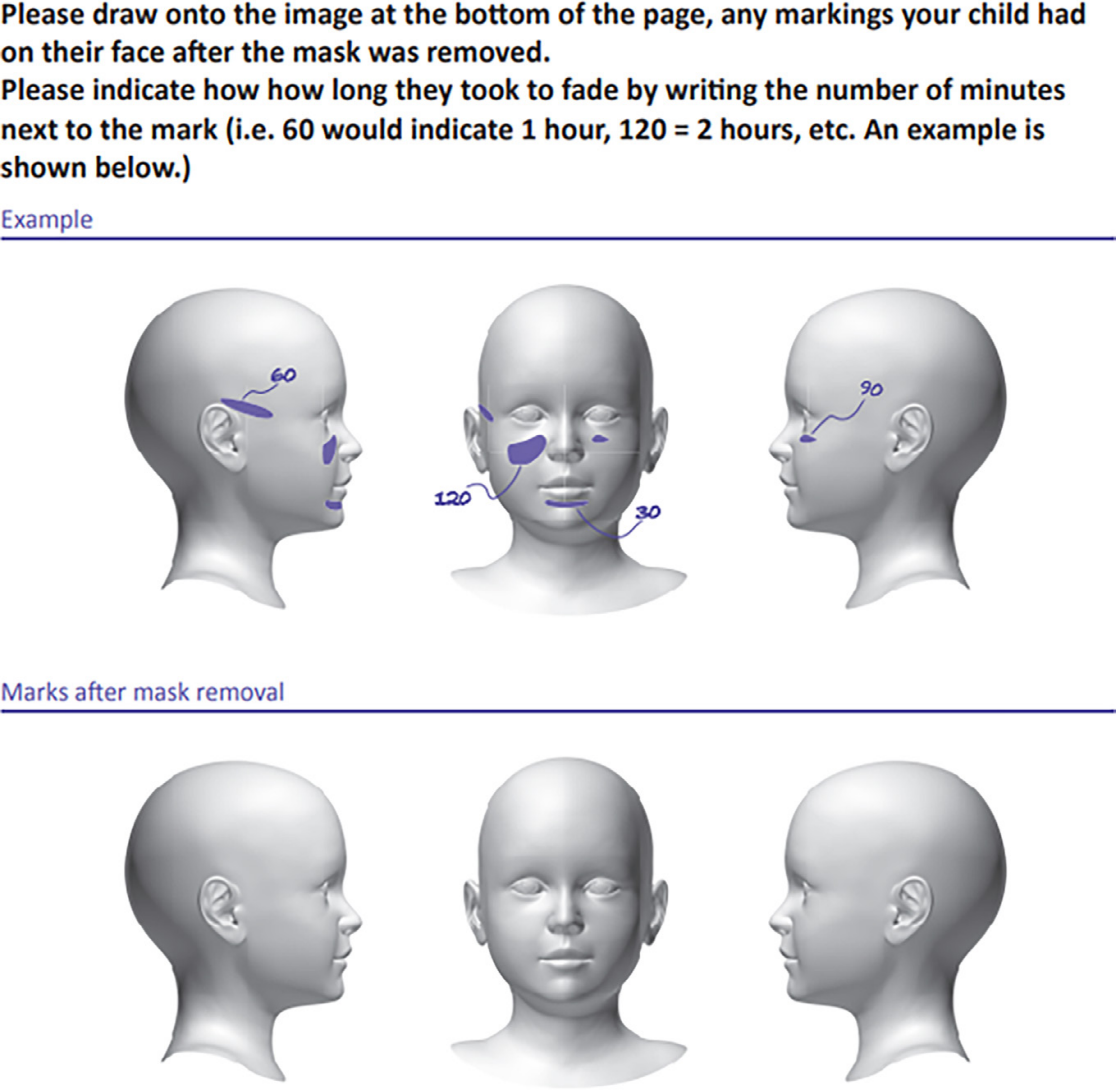
Section of diary card that asks the parent to record facial skin markings.

A diary card was used (figure 1) with pre-prepared face illustrations that allowed parents to draw areas of facial skin marks. The author MB counted the number of marks drawn on the pre-prepared face illustrations, converting the drawings into more usable quantitative data.

Subjective discomfort was measured using a numerical 1-point to 7-point Likert scale and noted on a diary card each morning; the discomfort scale was rated by parents on their child’s behalf. It was intended that the child and parent would complete the diary card (including the Likert scale) together in cases where the participant was able to contribute.

Ventilation efficacy was measured using 4% Oxygen Desaturation Index (ODI_4_) via pulse oximetry (Masimo, Radical 7); sleep quality was measured using Actual Wake % via actigraphy (MotionWatch 8, CamNTech Ltd).

Sleep data were scored using AASM standards (minimum 4 hours of consecutive data). Continuous variables were presented as median differences with IQR. No formal statistical analysis on the data was undertaken because of the very small sample size.

## Results

Of nine participants recruited, three were ineligible either due to inability to tolerate any mask or due to their facial dimensions being outside the size range available for this study. One withdrew due to medical deterioration prior to data collection. Five participants trialled the custom mask and custom headgear, age range 2–15 years, 1 female:4 males; medical indications: cerebral palsy, spinal muscular atrophy (SMA) type 1, SMA type 2, infantile Refsum disease and obstructive sleep apnoea.

Participant 2 completed three study nights only. On night four, he removed the custom setup prior to the 4-hour study duration due to nasal bridge soreness. Parents chose to not complete further study nights. Participant 5 preferred his standard mask with the custom headgear after trialling the custom mask for one night. On this night (night 3 of 4), he wore the mask for less than the pre-determined 4-hour minimum study duration. This night was excluded from the analysis.

**Figure 2:**
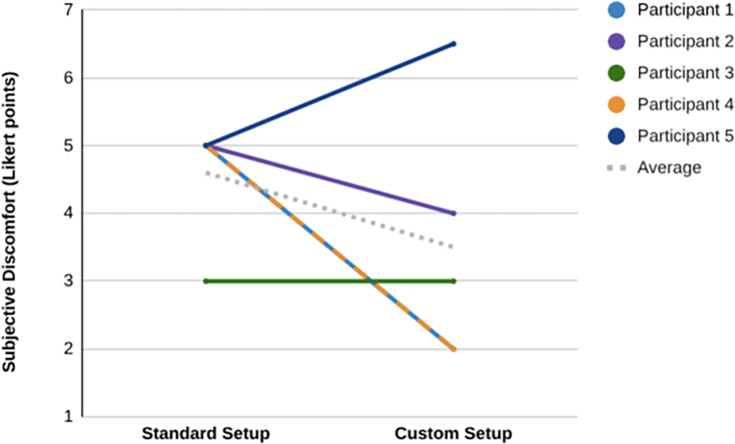
Difference in Subjective Discomfort score (Likert points) between standard mask/headgear and custom mask/headgear. Subjective discomfort was rated from 1 (very comfortable) to 7 (very uncomfortable). Colour-coded lines indicate individual changes for five participants. The grey dotted line indicates the overall mean difference.

Median subjective discomfort was rated by parents for all included participants as it was not possible for the children to contribute meaningfully. Scores rated from 1 (very comfortable) to 7 (very uncomfortable), as presented in figure 2. With standard mask/headgear the median score was 5 (IQR 0); with the custom mask/headgear, the median score was 3 (IQR 2).

The median number of facial skin marks with standard mask/headgear was 1.5 (IQR 3) and with the custom mask/headgear was 1 (IQR 0).

Median ODI_4_ with standard mask/headgear was 0.76 (IQR 0.8), and with the custom mask/headgear was 0.77 (IQR 0.71).

Median Actual Wake % with standard mask/headgear was 2.95 (IQR 2.31) and with the custom mask/headgear was 3.70 (IQR 4.85).

Results for all outcome measures are presented in table 1.

**Table 1:** Findings for each variable for participants with the custom setup compared with the standard setup

Variable (median; Q1-Q3; IQR)	Standard setup	Custom setup	Difference = (custom setup) - (Standard Setup)
Subjective discomfort	5 (5-5; 0)	3 (2-4; 2)	−2 (−3-−1; 2)
Facial skin marks	1.5 (1-4; 3)	1 (1-1; 0)	−0.5 (0-−3; −3)
ODI_4_	0.76 (0.54–1.34; 0.80)	0.77 (0.60–1.31; 0.71)	0.01 (0.06-−0.03; −0.09)
Actual wake %	2.95 (2.80–5.11; 2.31)	3.70 (1.80–6.65; 4.85)	0.75 (−1.0-1.54; 2.54)

ODI_4_, 4% oxygen desaturation index.

## Discussion

This is the first study to report performance and clinical efficacy of a custom 3D-printed NIV mask with integrated modular headgear compared with a standard commercial mask and headgear in paediatric patients using NIV. Discomfort, difficulties with adherence and ventilator inefficiencies due to suboptimal fit of commercially available masks for NIV are well-recognised complications of long-term ventilation.^5^ The results in this small cohort are encouraging, indicating that 3D designed and printed customised NIV mask and headgear may be a promising alternative in terms of comfort, facial skin marks, ODI_4_ and sleep disturbance, although it was not appropriate to carry out any statistical analysis due to the small sample size.

In this rapidly growing research field, studies of NIV mask customisation generally indicate positive outcomes in terms of comfort, air leak and pressure applied to the skin in bench testing, healthy volunteers and patient groups. Studies using patient participants have focused on adults using long-term ventilation or children using NIV in an acute setting for respiratory failure.^7^ There have been three single-case paediatric studies reported to date^9–11^ and no previous study has evaluated headgear in addition to a custom-made mask. This pilot study is the first to test custom-made 3D-printed masks as well as custom-made headgear to evaluate comfort as well as ventilator efficacy, facial soreness and sleep quality in children requiring NIV.

The small study size is a limitation that reduces the reliability of the results and thus they should be interpreted with caution. No formal statistical analysis was carried out, given the small sample size. The short study duration may have misrepresented differences, and the participants were recruited from a single centre. We acknowledge that a cross-over design might have been preferable although, as it was not feasible to blind the participants, parents or investigators to the mask type, we do not believe that allocation order would have had a significant impact but would consider this in a future study. We acknowledge that lack of blinding introduces the potential for bias, particularly for subjective outcomes such as comfort. Parental reporting of skin markings in diary records may similarly be influenced by knowledge of whether the custom mask and headgear are in use. However, the primary aim of the customisation is to improve tolerance of the mask, which is subject to an individual’s perception of comfort. It was intended that the child and parent would complete the diary card (including the Likert scale) together in cases where the participant was able to contribute, but in future studies, a face scale will be considered. All measures were found to improve, indicating that a larger study would be justified to refine the design further and definitively evaluate the clinician and cost efficiency outcomes. Future trials could also include specific comparisons of headgear separately from the mask.

Customisation of NIV masks using 3D printing may provide a valuable solution for this current unmet medical need although important gaps in knowledge remain. In this evolving landscape, materials’ biocompatibility is yet to be established for many flexible additive manufacturing options and further work to investigate cost-effectiveness of a custom-made approach compared with commercial masks is needed before recommendations for clinical implementation will be viable.

## Data Availability

Data sharing not applicable as no datasets generated and/or analysed for this study.
